# *CryoCrane*: an open-source GUI for analyzing cryo-EM screening data sets

**DOI:** 10.1107/S2053230X25000081

**Published:** 2025-01-13

**Authors:** Jakob Ruickoldt, Petra Wendler

**Affiliations:** a Institute for Biochemistry and Biology University of Potsdam Am Neuen Palais 10 14469Potsdam Germany; European Molecular Biology Laboratory, France

**Keywords:** cryo-EM, sample optimization, grid screening, cryo-EM data collection

## Abstract

Screening of cryo-EM samples is essential for the generation of high-resolution cryo-EM structures. Often, it is cumbersome to correlate the appearance of specific grid squares and micrograph quality. Here, *CryoCrane* (*C*o*r*relate *a*tlas a*n*d *e*xposures), a visualization tool for cryo-EM screening data, is presented. It aims to provide an intuitive way to visualize micrographs and to speed up data analysis.

## Abstract 2

*CryoCrane* is a GUI which allows rapid analysis of cryo-EM screening data and supervision of data collections.

## 1. Introduction

The optimization of sample preparation is crucial for the success of high-resolution cryo-electron microscopy (cryo-EM) studies (Cheng *et al.*, 2015 ▸; Dobro *et al.*, 2010 ▸; Thompson *et al.*, 2016 ▸; Passmore & Russo, 2016 ▸). The thickness and the quality of the vitrified sample depend on the sample application, blotting parameters, humidity and protein concentration, to name but a few. Screening of one grid takes from a few hours to a day depending on the specimen holder and the microscope setup. In difficult samples, ice quality and sample integrity can vary within one grid and users have to learn to discriminate areas suitable for data collection from those that are unusable. This process requires taking images in different areas of the grid, which then have to be manually assigned to areas in the overview image (atlas) using time tags. This process is rather cumbersome and time-consuming. Screening of several grid squares of a sample grid is necessary to find those areas in which the ice thickness, ice quality and particle distribution are optimal. To accelerate this process, we have developed a graphical user interface which allows visual exploration of the summed images and the atlas of a sample grid. This can reduce the time needed for grid assessment and for finding optimal ice quality for high-resolution data collection.

## 2. Methods

**Figure 1:**
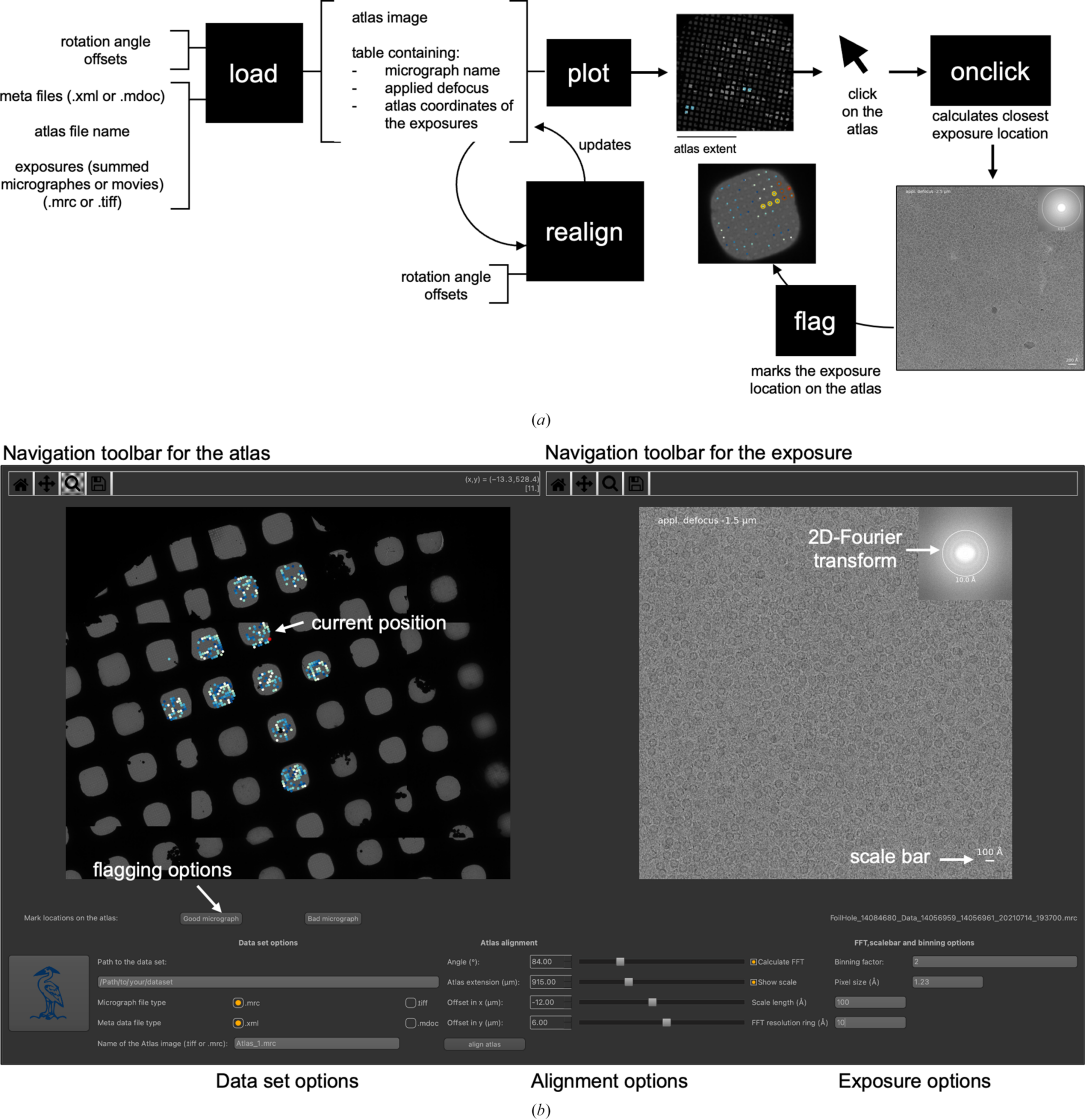
Overview of the workflow and GUI of *CryoCrane*. (*a*) Workflow of *CryoCrane*. The function ‘load’ takes the metadata and image files, the atlas file, the rotation angle and the offsets as inputs and outputs a table containing the (atlas) coordinates of each exposure, the applied defocus and the file name. The coordinates can be fine-tuned with the ‘realign’ function. The ‘plot’ function then displays the atlas image and overlays the exposure locations. Upon clicking on the atlas image the ‘onclick’ function displays the exposures according to the user settings. The user then has the possibility of flagging a certain exposure position as good or bad with the ‘flag’ function. (*b*) Overview of the GUI. The two upper panels are the heart of the GUI. The left panel shows the atlas image and the locations of the exposures, while the right panel shows the respective exposure upon clicking on a specific location in the left panel. The uppermost panels are navigation toolbars for the plotted images allowing zooming, panning and saving of the image. The lower panels harbor the settings. Clicking on the *CryoCrane* logo will attempt to plot the atlas and exposure locations. The user has to specify the paths and file types. Furthermore, the central panel contains the parameters for aligning atlas and stage coordinates, while in the leftmost panel the parameters for FFT, scale bars and binning can be set.

The program was written in Python and relies on the *NumPy* (Harris *et al.*, 2020 ▸), *matplotlib* (Hunter, 2007 ▸), *pandas* (McKinney, 2010 ▸), *mrcfile* (Burnley *et al.*, 2017 ▸), *pyqt*5 and *pathlib* packages. The program is designed for the analysis of data sets recorded with *EPU* (ThermoFisher Scientific) or *SerialEM* (Mastronarde, 2003 ▸). Support for *Leginon* (Cheng *et al.*, 2021 ▸) data sets will be added in the future. The user has to enter the path to the folder containing the data set and the name of the atlas image or the path to the atlas image (if it is not located in the data-set directory; Fig. 1 ▸). After pressing the *CryoCrane* button the data set will be read and plotted. In the current setup the program searches the path supplied by the user for an atlas file (.mrc or .tiff; specified by the user) that is created from several low-magnification images by the data-collection software. Alternatively, one can also supply the absolute path to the atlas image. Afterwards, it collects the stage coordinates and the applied beam shift of the exposures from the meta files (.xml or .mdoc) by searching for specific strings in the files that are output by *EPU* or *SerialEM*. The coordinates (which are the stage coordinates) are transformed to the atlas coordinate system. The parameters for this transformation depend on the microscope and can be adjusted by the user. This transformation uses the formula
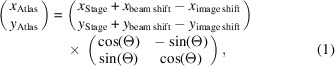
where Θ is the rotation angle, *x*_beam shift_ (or *y*_beam shift_) is the applied beam shift and *x*_image shift_ (or *y*_image shift_) is a constant coordinate offset. The locations and the micrograph files are then stored in a table and plotted. The user can zoom, pan and save the atlas and exposure with the navigation toolbar above the plots. The parameters for the transformation of the stage coordinates to the atlas coordinate system can be entered right next to the path-input field. These need to be adjusted for every microscope and data set. The values fitting a microscope can be stored in the source code as defaults (after the #Default values line in the source code). Aligning the stage and atlas coordinates works best if one first determines the rotation angle and subsequently the extent of the atlas by comparing the hole spacing with that of the data. These two parameters will be similar for data sets recorded using the same microscope. In the following, the *x* and *y* shifts can be adjusted, which requires fine-tuning for every data set. The foil holes are colored according to the applied defocus. Specific micrographs can be accessed by the user by clicking on a location on the atlas image. The program will then show the micrograph of the nearest foil hole. Furthermore, a 2D Fourier transform of the micrograph and a scale bar can be plotted, if requested (Fig. 1 ▸). *CryoCrane* works best with the summed micrographs output by *EPU*. It can also read movies and sum them on the fly. However, depending on the network speed this may become prohibitively slow.

The program can be installed by following the installation instructions given in the Github repository (https://github.com/jruickoldt/CryoCrane/). The installation procedure will create a new Python environment named ‘CryoCrane’ and requires *Miniconda* (https://docs.anaconda.com/miniconda/) to be installed. The program can be started by changing the directory to that containing CryoCrane.py, activating the *CryoCrane* environment and typing python3 CryoCrane.py. The installation has been tested on MacOS Sonoma and Windows 11 (using Anaconda Navigator).

## 3. Results

The GUI of *CryoCrane* shows the atlas file and the selected exposure side by side and enables the user to zoom into grid squares of their choice and assess the quality of all foil holes within minutes. The evaluation is simplified by displaying the applied defocus during data collection and a simplified 2D Fourier transform based on the summed movie frames of the exposure. A scale bar allows size estimation of the sample.

### 3.1. Example: analysis of an UltraAuFoil grid

**Figure 2:**
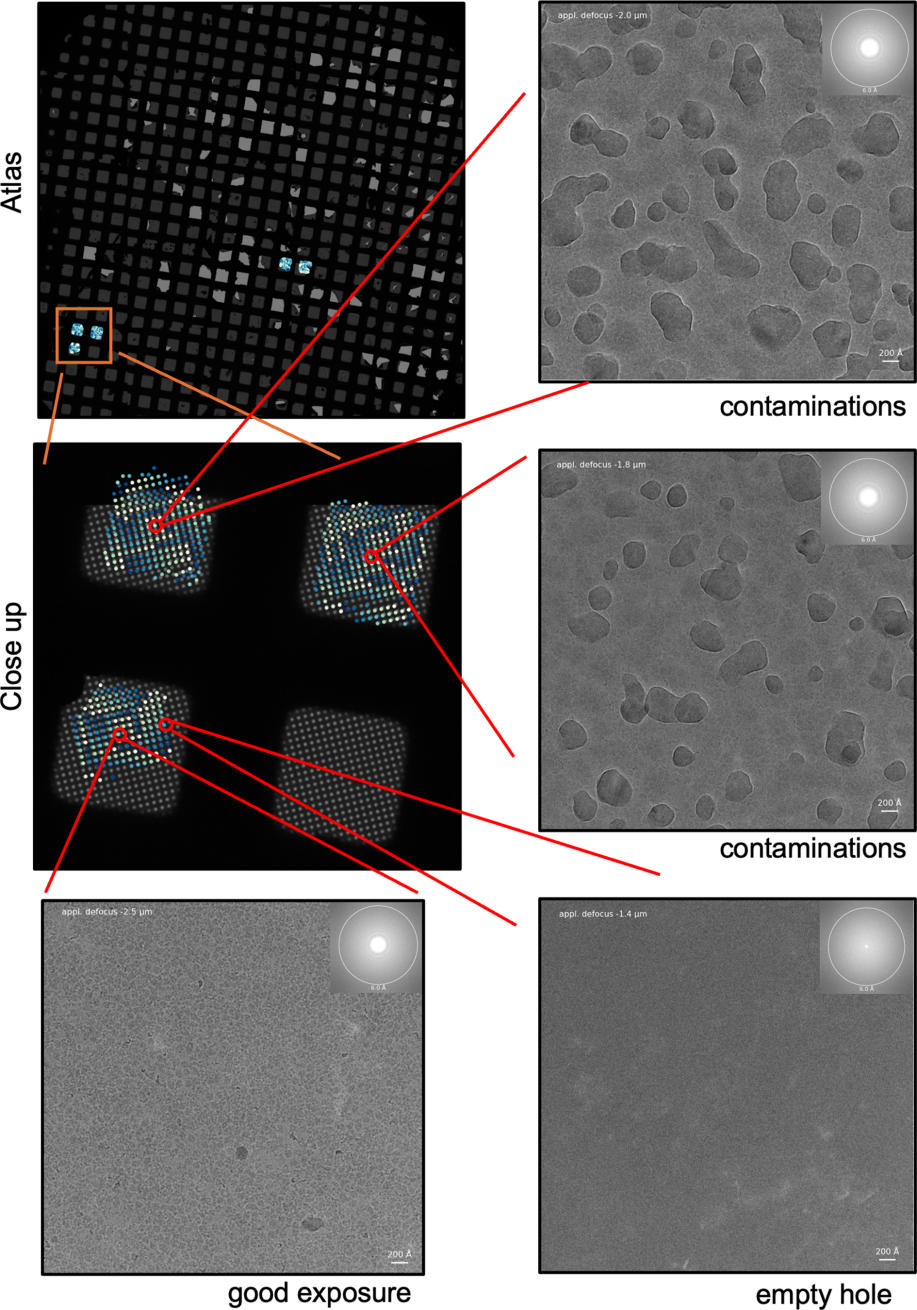
Usage example: screening of an UltraAuFoil grid. The atlas and the locations of the exposures are shown in the upper left corner and a close-up of specific squares is shown right below. The micrographs from the two upper squares in this close-up were mostly contaminated, while those in the lower square were not. However, in the lower square only the central holes were filled. The micrographs were recorded at magnification of 92 000 on a Talos F200C equipped with a Falcon 3 camera and a dose of ∼50 e^−^ Å^−2^.

Data collection from UltraAuFoil grids can be tedious as empty and filled foil holes cannot easily be distinguished (unless the microscope has an energy filter). With *CryoCrane* it is rather simple to quickly analyze which foil holes are suitable for data collection (Fig. 2 ▸). In this case, *CryoCrane* was used to adjust a data collection manually on the fly and direct it to more promising areas. The analysis showed that the best micrographs were obtained from ice patches found in the middle of the grid square, while the holes at the rim were empty. Furthermore, some squares had contaminated areas, which should be avoided. If a data-collection session is analyzed on the fly these squares can be skipped.

## 4. Conclusion

We present a GUI for the easy analysis of cryo-EM screening data. The software enables cryo-EM users to learn which grid areas are most promising for high-resolution data collection. It can be improved by further automation. Therefore, we plan to implement a machine-learning model to automatically judge the quality of the micrographs. This would allow even more rapid identification of the best areas for data collection on a grid. Later, this might be connected to a target-identification algorithm that only collects data from promising areas of a grid, reducing the unnecessary use of storage capacity by unusable exposures and the waste of measurement time.

## Data Availability

The source code is freely available on Github (https://github.com/jruickoldt/CryoCrane/).
